# Supplementary material for: Discovery and application of insertion-deletion (INDEL) polymorphisms for QTL mapping of early life-history traits in Atlantic salmon
Source: BMC Genomics. 2010 Mar 8;11:156. doi: 10.1186/1471-2164-11-156 (PMC2838853; doi:10.1186/1471-2164-11-156)
Supplement: Additional file 2 — Information on developed 76 locus single-run INDEL panel in Atlantic salmon. Information on fluorescence labeling, primer concentrations, PCR pooling and links to alignments, INDEL motifs and GENESCAN (Burge and Karlin 1997) predictions of genes/exons are available in html format. [file 1471-2164-11-156-S2.ZIP › Additionalfile2/snpsummary3270.html]

```
Cluster 674 Contig 2

prev  Summary    Contig List  next
```

Size of Consensus sequence = 1090

Number of sequences = 49

Minimum redundancy = 6

Key

A gi|83640362|gb|DW006952.1|DW006952 SGP296450 Atlantic salmon Brain cDNA library Salmo salar cDNA clone HJ4-3681 5', mRNA sequence  
B gi|117472700|gb|EG804919.1|EG804919 EST\_ssal\_evd\_5746 ssalevd thymus Salmo salar cDNA Salmo salar cDNA clone ssal\_evd\_506\_140\_fwd 3', mRNA sequence  
C gi|117439011|gb|EG771234.1|EG771234 EST\_ssal\_evd\_3247 ssalevd thymus Salmo salar cDNA Salmo salar cDNA clone ssal\_evd\_502\_366\_fwd 3', mRNA sequence  
D gi|117496772|gb|EG828989.1|EG828989 EST\_ssal\_eve\_41382 ssaleve thyroid Salmo salar cDNA Salmo salar cDNA clone ssal\_eve\_556\_112\_fwd 3', mRNA sequence  
E gi|117853554|gb|EG926250.1|EG926250 EST\_ssal\_evf\_28782 ssalevf mixed\_tissue Salmo salar cDNA Salmo salar cDNA clone ssal\_evf\_538\_065\_fwd 3', mRNA sequence  
F gi|117439081|gb|EG771304.1|EG771304 EST\_ssal\_evd\_46958 ssalevd thymus Salmo salar cDNA Salmo salar cDNA clone ssal\_evd\_563\_037\_fwd 3', mRNA sequence  
G gi|117864460|gb|EG937156.1|EG937156 EST\_ssal\_evf\_21009 ssalevf mixed\_tissue Salmo salar cDNA Salmo salar cDNA clone ssal\_evf\_526\_242\_fwd 3', mRNA sequence  
H gi|117523073|gb|EG854800.1|EG854800 EST\_ssal\_eve\_15513 ssaleve thyroid Salmo salar cDNA Salmo salar cDNA clone ssal\_eve\_521\_029\_fwd 3', mRNA sequence  
I gi|117845776|gb|EG918472.1|EG918472 EST\_ssal\_evf\_57522 ssalevf mixed\_tissue Salmo salar cDNA Salmo salar cDNA clone ssal\_evf\_577\_067\_fwd 3', mRNA sequence  
J gi|117568709|gb|EG889685.1|EG889685 EST\_ssal\_evf\_47617 ssalevf mixed\_tissue Salmo salar cDNA Salmo salar cDNA clone ssal\_evf\_563\_251\_fwd 3', mRNA sequence  
K gi|117551389|gb|EG882144.1|EG882144 EST\_ssal\_eve\_25290 ssaleve thyroid Salmo salar cDNA Salmo salar cDNA clone ssal\_eve\_534\_160\_fwd 3', mRNA sequence  
L gi|117430654|gb|EG762878.1|EG762878 EST\_ssal\_sjb\_3869 ssalsjb mixed\_tissue Salmo salar cDNA Salmo salar cDNA clone ssal\_sjb\_010\_238\_fwd 3', mRNA sequence  
M gi|117550532|gb|EG881817.1|EG881817 EST\_ssal\_eve\_24996 ssaleve thyroid Salmo salar cDNA Salmo salar cDNA clone ssal\_eve\_534\_011\_fwd 3', mRNA sequence  
N gi|117854288|gb|EG926984.1|EG926984 EST\_ssal\_evf\_29443 ssalevf mixed\_tissue Salmo salar cDNA Salmo salar cDNA clone ssal\_evf\_539\_021\_fwd 3', mRNA sequence  
O gi|117491205|gb|EG823422.1|EG823422 EST\_ssal\_evd\_25060 ssalevd thymus Salmo salar cDNA Salmo salar cDNA clone ssal\_evd\_532\_226\_fwd 3', mRNA sequence  
P gi|117501420|gb|EG833436.1|EG833436 EST\_ssal\_eve\_45385 ssaleve thyroid Salmo salar cDNA Salmo salar cDNA clone ssal\_eve\_561\_283\_fwd 3', mRNA sequence  
Q gi|117563993|gb|EG884969.1|EG884969 EST\_ssal\_evf\_43373 ssalevf mixed\_tissue Salmo salar cDNA Salmo salar cDNA clone ssal\_evf\_557\_369\_fwd 3', mRNA sequence  
R gi|117568707|gb|EG889683.1|EG889683 EST\_ssal\_evf\_47616 ssalevf mixed\_tissue Salmo salar cDNA Salmo salar cDNA clone ssal\_evf\_563\_251\_rev 5', mRNA sequence  
S gi|117563071|gb|EG884047.1|EG884047 EST\_ssal\_evf\_42540 ssalevf mixed\_tissue Salmo salar cDNA Salmo salar cDNA clone ssal\_evf\_556\_326\_fwd 3', mRNA sequence  
T gi|117821781|gb|EG894477.1|EG894477 EST\_ssal\_evf\_51931 ssalevf mixed\_tissue Salmo salar cDNA Salmo salar cDNA clone ssal\_evf\_569\_200\_fwd 3', mRNA sequence  
U gi|117821782|gb|EG894478.1|EG894478 EST\_ssal\_evf\_51932 ssalevf mixed\_tissue Salmo salar cDNA Salmo salar cDNA clone ssal\_evf\_569\_200\_rev 5', mRNA sequence  
V gi|117540097|gb|EG871542.1|EG871542 EST\_ssal\_eve\_32580 ssaleve thyroid Salmo salar cDNA Salmo salar cDNA clone ssal\_eve\_544\_087\_fwd 3', mRNA sequence  
W gi|117550533|gb|EG881818.1|EG881818 EST\_ssal\_eve\_24997 ssaleve thyroid Salmo salar cDNA Salmo salar cDNA clone ssal\_eve\_534\_011\_rev 5', mRNA sequence  
X gi|117863058|gb|EG935754.1|EG935754 EST\_ssal\_evf\_19746 ssalevf mixed\_tissue Salmo salar cDNA Salmo salar cDNA clone ssal\_evf\_524\_361\_fwd 3', mRNA sequence  
Y gi|117430765|gb|EG762989.1|EG762989 EST\_ssal\_sjb\_3870 ssalsjb mixed\_tissue Salmo salar cDNA Salmo salar cDNA clone ssal\_sjb\_010\_238\_rev 5', mRNA sequence  
Z gi|117551387|gb|EG882142.1|EG882142 EST\_ssal\_eve\_25289 ssaleve thyroid Salmo salar cDNA Salmo salar cDNA clone ssal\_eve\_534\_160\_rev 5', mRNA sequence  
a gi|117540098|gb|EG871543.1|EG871543 EST\_ssal\_eve\_32581 ssaleve thyroid Salmo salar cDNA Salmo salar cDNA clone ssal\_eve\_544\_087\_rev 5', mRNA sequence  
b gi|117850111|gb|EG922807.1|EG922807 EST\_ssal\_evf\_1884 ssalevf mixed\_tissue Salmo salar cDNA Salmo salar cDNA clone ssal\_evf\_006\_267\_rev 5', mRNA sequence  
c gi|117563992|gb|EG884968.1|EG884968 EST\_ssal\_evf\_43372 ssalevf mixed\_tissue Salmo salar cDNA Salmo salar cDNA clone ssal\_evf\_557\_369\_rev 5', mRNA sequence  
d gi|117850113|gb|EG922809.1|EG922809 EST\_ssal\_evf\_1885 ssalevf mixed\_tissue Salmo salar cDNA Salmo salar cDNA clone ssal\_evf\_006\_267\_fwd 3', mRNA sequence  
e gi|117859190|gb|EG931886.1|EG931886 EST\_ssal\_evf\_18429 ssalevf mixed\_tissue Salmo salar cDNA Salmo salar cDNA clone ssal\_evf\_523\_064\_rev 5', mRNA sequence  
f gi|117859201|gb|EG931897.1|EG931897 EST\_ssal\_evf\_18430 ssalevf mixed\_tissue Salmo salar cDNA Salmo salar cDNA clone ssal\_evf\_523\_064\_fwd 3', mRNA sequence  
g gi|117864459|gb|EG937155.1|EG937155 EST\_ssal\_evf\_21008 ssalevf mixed\_tissue Salmo salar cDNA Salmo salar cDNA clone ssal\_evf\_526\_242\_rev 5', mRNA sequence  
h gi|117492672|gb|EG824889.1|EG824889 EST\_ssal\_evd\_26381 ssalevd thymus Salmo salar cDNA Salmo salar cDNA clone ssal\_evd\_534\_144\_fwd 3', mRNA sequence  
i gi|117496773|gb|EG828990.1|EG828990 EST\_ssal\_eve\_41383 ssaleve thyroid Salmo salar cDNA Salmo salar cDNA clone ssal\_eve\_556\_112\_rev 5', mRNA sequence  
j gi|117472699|gb|EG804918.1|EG804918 EST\_ssal\_evd\_5745 ssalevd thymus Salmo salar cDNA Salmo salar cDNA clone ssal\_evd\_506\_140\_rev 5', mRNA sequence  
k gi|117439080|gb|EG771303.1|EG771303 EST\_ssal\_evd\_46957 ssalevd thymus Salmo salar cDNA Salmo salar cDNA clone ssal\_evd\_563\_037\_rev 5', mRNA sequence  
l gi|117523074|gb|EG854801.1|EG854801 EST\_ssal\_eve\_15514 ssaleve thyroid Salmo salar cDNA Salmo salar cDNA clone ssal\_eve\_521\_029\_rev 5', mRNA sequence  
m gi|117438056|gb|EG770279.1|EG770279 EST\_ssal\_evd\_46035 ssalevd thymus Salmo salar cDNA Salmo salar cDNA clone ssal\_evd\_561\_319\_fwd 3', mRNA sequence  
n gi|117845775|gb|EG918471.1|EG918471 EST\_ssal\_evf\_57521 ssalevf mixed\_tissue Salmo salar cDNA Salmo salar cDNA clone ssal\_evf\_577\_067\_rev 5', mRNA sequence  
o gi|117863057|gb|EG935753.1|EG935753 EST\_ssal\_evf\_19745 ssalevf mixed\_tissue Salmo salar cDNA Salmo salar cDNA clone ssal\_evf\_524\_361\_rev 5', mRNA sequence  
p gi|117853555|gb|EG926251.1|EG926251 EST\_ssal\_evf\_28783 ssalevf mixed\_tissue Salmo salar cDNA Salmo salar cDNA clone ssal\_evf\_538\_065\_rev 5', mRNA sequence  
q gi|117491203|gb|EG823420.1|EG823420 EST\_ssal\_evd\_25059 ssalevd thymus Salmo salar cDNA Salmo salar cDNA clone ssal\_evd\_532\_226\_rev 5', mRNA sequence  
r gi|117438057|gb|EG770280.1|EG770280 EST\_ssal\_evd\_46036 ssalevd thymus Salmo salar cDNA Salmo salar cDNA clone ssal\_evd\_561\_319\_rev 5', mRNA sequence  
s gi|117439022|gb|EG771245.1|EG771245 EST\_ssal\_evd\_3248 ssalevd thymus Salmo salar cDNA Salmo salar cDNA clone ssal\_evd\_502\_366\_rev 5', mRNA sequence  
t gi|117492671|gb|EG824888.1|EG824888 EST\_ssal\_evd\_26380 ssalevd thymus Salmo salar cDNA Salmo salar cDNA clone ssal\_evd\_534\_144\_rev 5', mRNA sequence  
u gi|117501421|gb|EG833437.1|EG833437 EST\_ssal\_eve\_45386 ssaleve thyroid Salmo salar cDNA Salmo salar cDNA clone ssal\_eve\_561\_283\_rev 5', mRNA sequence  
v gi|29311677|gb|CB500451.1|CB500451 ssalra022049 head Salmo salar cDNA, mRNA sequence  
w gi|29311675|gb|CB500449.1|CB500449 ssalra022046 head Salmo salar cDNA, mRNA sequence

4 SNPs detected

A B C D E F G H I J K L M N O P Q R S T U V W X Y Z a b c d e f g h i j k l m n o p q r s t u v w  cosegregation weighted

490 . T C T T C T T T T T C T T C T T T T T T T . T C . T . T T T T T C . . . T C T T T C C C C . . .   1/4 19.90
944 . . . . . . . T . C C C - - - C C C - - - C - - C C C C C C C C C C C C C - - - - - - - C C C C C   2/4 41.84
945 . . . . . . . A . A A A - - - A A A - - - A - - A A A A A A A A A A A A A - - - - - - - A A A A A   1/4 20.92
946 . . . . . . . A . G G G - - - G G G - - - G - - G G G G G G G G G G G G G - - - - - - - G G G G G   2/4 41.84
